# Heart Failure Care Delivery in the COVID-19 Era: The Patients’ Perspective

**DOI:** 10.3390/healthcare9030245

**Published:** 2021-03-01

**Authors:** Meg Fraser, Melinda Mutschler, Christie Newman, Kerry Sackman, Batul Mehdi, Linda Wick, Sue Duval, Gary S. Francis, Tamas Alexy

**Affiliations:** Department of Medicine, Division of Cardiology, University of Minnesota, Minneapolis, MN 55455, USA; mmutschl10@umphysicians.umn.edu (M.M.); cnewman10@umphysicians.umn.edu (C.N.); ksackman10@umphysicians.umn.edu (K.S.); bmehdi10@umphysicians.umn.edu (B.M.); lwick1@fairview.org (L.W.); sueduval@umn.edu (S.D.); franc354@umn.edu (G.S.F.); alexy001@umn.edu (T.A.)

**Keywords:** COVID-19, heart failure care delivery, telemedicine, patient survey

## Abstract

*Purpose*: The SARS-CoV-2 pandemic is changing healthcare delivery around the world with hospital systems experiencing a dramatic decline in patient volumes. Surveying our center’s heart failure (HF) clinic population, we aimed to understand our patients’ perception of coronavirus disease 2019 (COVID-19) and care delivery preferences. *Methods*: Patients with chronic HF presenting either in-person or virtually were approached to complete a ten question, anonymous, voluntary survey. Acutely decompensated patients and heart transplant recipients were excluded. *Results*: 109 patients completed the survey. Average age was 62 ± 14 years, 67% were male, and 59% had HF with reduced ejection fraction (HFrEF). Overall, patients were worried about contracting COVID-19 and believed they were prone to more severe infection given their underlying HF. However, they were not hesitant to initiate healthcare contact for symptoms and preferred in-person appointments over virtual visits. Although the difference did not reach statistical significance, female patients and those with HF with preserved ejection fraction (HFpEF) were more concerned. *Conclusions*: Patients with HF are concerned about their increased risk of contracting COVID-19. However, they are actively seeking healthcare contact and prefer in-person over virtual visits.

## 1. Introduction

Coronavirus disease 2019 (COVID-19) was declared a pandemic by the World Health Organization on 11 March 2020. Community mitigation measures contributed to a significant decline in patient-initiated healthcare contacts, including emergency room (ER) visits, outpatient appointments, and hospital admissions, even for life-threatening conditions [1]. However, healthcare systems quickly recognized that patients require continued, safe access to medical care, especially those with chronic conditions, such as heart failure (HF) [2]. Most commercial payers and the Centers for Medicare and Medicaid Services (CMS) waived restrictions on telehealth visits, enabling continued care delivery using telephone and real-time video platforms [3,4]. Virtual encounters almost immediately became an integral part of outpatient management. Benefits of virtual visits stem from the abolished SARS-CoV-2 exposure risk in the clinic setting, reduced patient stress and anxiety, continued patient-provider partnership, and lower hospital admission rates, preserving beds for the critically ill [5,6]. Many reports have been published on the widespread benefits of this care model in the setting of the pandemic from the providers’ perspective, although it is not without limitations. Accurate vital signs can rarely be obtained, physical examination is limited and challenging, and difficulties related to the use of virtual technology are not uncommon. In addition, our understanding of the patients’ perspective remains limited. It is unclear how they have adapted to this rapid change, especially the elderly and chronically ill, how effective virtual care delivery is in their opinion, and whether they preferred in-person visits with their providers. Our hypothesis was that patients with HF are worried to contract COVID-19. This would influence their likelihood to continue medical care for their chronic condition and increase their threshold to seek expert attention during acute exacerbation. Given the importance of in-person physical examination to assess volume status and organ perfusion, we further hypothesized that patients with HF prefer in-person visits with their providers over virtual care.

## 2. Materials and Methods

Adult patients presenting for in-person or virtual follow-up visits to the University of Minnesota Cardiomyopathy, Optimization, Rehabilitation, and Education (C.O.R.E.) clinic between August and October 2020 were eligible to participate. This clinic is staffed by advanced practice providers under the supervision of advanced heart failure cardiologists. Patients with acute HF decompensation requiring hospital admission and heart transplant recipients were excluded from the study.

**Figure 1:**
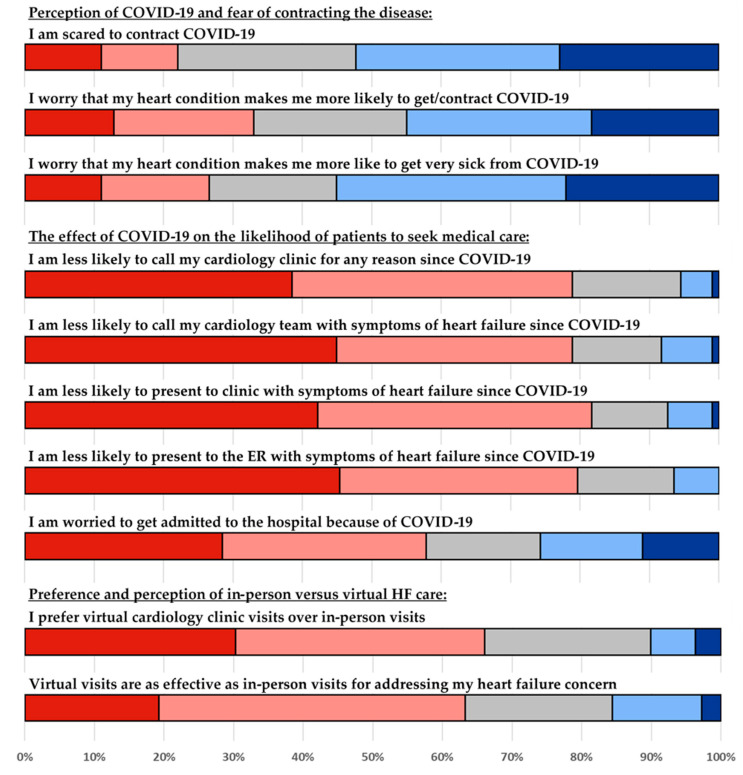
Survey questions and responses shown on a Likert scale with a range of −2 to 2. Strongly disagree (−2, red), Disagree (−1, pink), Neither agree nor disagree (0, grey), Agree (1, light blue), Strongly agree (2, dark blue).

Completing the survey took less than 5 min, and participation was voluntary. Patients were approached during their appointment and asked to voluntarily complete a 10-question survey utilizing a 5-point Likert scale with possible responses ranging from strongly disagree (−2) to strongly agree (+2) (Figure 1). Given the unique circumstances presented by the COVID-19 pandemic, a well-validated or widely utilized survey was not available. As such, we decided to pose questions around three general themes: (1) patient’s perception of COVID-19 and their fear of contracting the disease (questions 1–3); (2) the effect of COVID-19 on their likelihood to seek medical care (questions 4–8); (3) patients’ preference and perception regarding in-person versus virtual HF care (questions 9–10). Reliability was assessed using Cronbach’s alpha, with values higher than 0.7 considered to indicate good internal consistency. In addition to the survey responses, basic demographic information was collected including age, gender, race, and HF type based on left ventricular ejection fraction (HF with reduced ejection fraction (HFrEF, <40%)), HF with midrange ejection fraction (HFmrEF, 40–49%), and HF with preserved ejection fraction (HFpEF, ≥50%)). The most recent echocardiogram or cardiac MRI study available to investigators was used to define HF type. The research team was blinded to any individually identifiable patient information and the date the survey was collected on. All data are reported in aggregate. Comparisons between patient subgroups were performed using two tailed t-test and a p-value less than 0.05 was considered statistically significant.

## 3. Results

109 patients completed the survey (Table 1). The questionnaire was found to have good internal consistency (10 items; alpha = 0.77). The average age of respondents was 62 ± 14 years, 73 (67%) were male, and 66 (60.5%) self-identified as white. Sixty-four patients (58.7%) had an ejection fraction (EF) < 40%, 9 (8.3%) had an EF of 40–49%, and 36 (33%) had preserved EF ≥ 50%. The cohort expressed that, overall, they were worried about contracting COVID-19 and believed that they are at elevated risk for severe infection owing to their underlying chronic HF (Table 2, Figure 1). Women and individuals with preserved EF appeared to be more concerned, but the difference did not reach statistical significance. When analyzed by age group, patients between 56 and 70 years were significantly more worried than the other age groups that HF made them more susceptible to COVID-19 (*p* < 0.05). Patients uniformly noted that the pandemic did not affect their willingness or likelihood to initiate healthcare contact for worsening HF symptoms. Responders were not hesitant to proceed with an outpatient clinic visit or evaluation in the ER. They were, however, slightly more reluctant to undergo hospital admission to receive treatment for HF exacerbation. Regardless of age, gender, race, or HF type, our survey indicated that patients with HF prefer in person visits with their providers and feel that virtual encounters are less effective to address their health-related concerns.

## 4. Discussion

The rapid emergence of the COVID-19 pandemic has prompted sweeping changes in healthcare delivery, particularly with telemedicine services experiencing unprecedented growth [7]. The routine use of virtual visits has enabled providers to continue uninterrupted services for patients with chronic conditions, such as HF. However, limited data are available on the patient’s perception of the pandemic, their likelihood to seek medical attention, and their visit preferences [8]. Our single-center survey of patients with chronic HF showed that, despite their fear of contracting COVID-19, they are likely to contact their provider or present for outpatient evaluation when experiencing worsening HF symptoms. However, they are slightly more reluctant to proceed with hospital admission. Our patients had a clear preference for in-person visits over virtual encounters. Despite our hypothesis that age may represent a barrier for telemedicine and that the elderly may prefer in-person encounters, the findings held true across all age groups. While our results for visit preference seemingly contrast national healthcare utilization data for worsening HF observed during the spring and summer of 2020, our survey was performed August through October 2020, several months into the pandemic. By this time, most clinics had reopened partially, and our strategies to mitigate COVID-19 exposure risk improved. Nevertheless, with the re-emerging virus surge, it is important to understand the preference and address the needs of our patients while delivering safe and efficient medical care. We acknowledge that this is a single-center study performed at a subspecialty clinic focusing on chronic HF. Findings may not be generalizable to other specialties or geographic areas. Approximately 65% of the surveys were collected during in-person encounters that may have slightly enriched for patients preferring in-person visits.

## 5. Conclusions

Patients with HF are concerned about their increased risk of contracting COVID-19 owing to their underlying disease. They are, however, not hesitant to initiate healthcare contact and preferred in-person encounters over virtual care, independent of age. The COVID-19 pandemic presents a constantly evolving situation, especially with the development of multiple vaccines. Harmonizing care delivery with patient preferences remains essential during these challenging times.

## Figures and Tables

**Table 1:** Demographics of the surveyed population.

Gender	*N*	%
Female	36	33%
Male	73	67%
Age Groups		
≤55 years	32	29.3%
56–70 years	46	42.2%
>70 years	31	28.5%
Race		
White	66	60.5%
African American	20	18.3%
Latino/Hispanic	3	2.8%
Asian	3	2.8%
Other/not stated	17	15.6%
Heart Failure Type		
HFpEF (EF ≥ 50%)	36	33.0%
HFmrEF (EF 40 - 49%)	9	8.3%
HFrEF (EF < 40%)	64	58.7%

HF, heart failure; HFpEF, HF with preserved ejection fraction; HFmrEF, HF with midrange ejection fraction; HFrEF, HF with reduced ejection fraction.

**Table 2:** Survey questions and distribution of responses. −2 = strongly disagree, −1 = disagree, 0 = neither disagree or agree, 1 = agree, 2 = strongly agree.

Question	Survey Question	Mean	Standard Deviation
Q1	I am frightened to contract COVID-19	0.4	1.3
Q2	I worry that my heart condition makes me more likely to get/contract COVID-19	0.2	1.3
Q3	I worry that my heart condition makes me more like to get very sick from COVID-19	0.4	1.3
Q4	I am less likely to call my cardiology clinic for any reason since COVID-19	−1.1	0.9
Q5	I am less likely to call my cardiology team with symptoms of heart failure since COVID-19	−1.1	1.0
Q6	I am less likely to present to clinic with symptoms of heart failure since COVID-19	−1.2	0.9
Q7	I am less likely to present to the Emergency Room (ER) with symptoms of heart failure since COVID-19	−1.2	0.9
Q8	I am reluctant to be admitted to the hospital because of COVID-19	−0.5	1.3
Q9	I prefer virtual cardiology clinic visits over in-person visits	−0.8	1.1
Q10	Virtual visits are as effective as in-person visits for addressing my heart failure concern	−0.6	1.0

## Data Availability

The data presented in this study are available within the article.
